# Characterization and purification of Algerian natural bentonite for pharmaceutical and cosmetic applications

**DOI:** 10.1186/s13065-021-00776-9

**Published:** 2021-09-01

**Authors:** Nabil Babahoum, Malek Ould Hamou

**Affiliations:** Mining Department of National Polytechnic School, 10 Avenue Hassen Badi BP 182 El Harrach, 16200 Algiers, Algeria

**Keywords:** Bentonite, Hammam Boughrara, Pharmacopeias, Excipient, Powders

## Abstract

**Introduction:**

Bentonitic clays from the Hammam Boughrara deposit in the Maghnia area (northwestern Algeria) were studied by mineralogical, chemical and physicochemical characterization to evaluate their potential suitability as raw and purified materials in pharmaceutical and cosmetic applications.

**Methodology:**

Natural bentonite was purified by Na+ ion exchange treatment combined with sedimentation techniques. Before use in the pharmaceutical industry, bentonite samples must be safe and conform to recommendations and directives of pharmacopeia. A set of technological tests were investigated with the samples, such as cation exchange capacity (CEC), specific surface area (SSA), swelling capacity (SC),sedimentation volume (SV) and viscosity, and mineralogical, chemical and microbial properties were also identified by X-ray fluorescence (XRF), X-ray diffraction (XRD) and scanning electron microscopy (SEM).

**Results:**

Mineralogical data proved that the raw bentonite is mainly composed of smectite and illite with small quantities of gangue minerals such quartz, feldspars (orthoclase and albite) and calcite**.** The purified bentonite matches the mineralogical properties of Wyoming bentonite as an international standard clay (deposits of high economic value). Quartz and feldspars were successfully eliminated in the absence of illite and calcite after beneficiation. Investigation of chemical analyses indicated that the contents of trace elements (particularly Pb and As) were below the more restrictive limits proposed by major pharmacopeias for raw and purified bentonite clay. For microbiological tests, the absence of Escherichia coli, Salmonella species, Staphylococcus aureus and Pseudomonas aeruginosa was confirmed. Moreover, we note that a high cation exchange capacity, large surface area, and good swelling capacity and sedimentation volume were also obtained for purified bentonite.

**Conclusion:**

In view of the fundamentals of major pharmacopoeias for the use of bentonite in pharmacies and considering the results obtained, we identified a pharmaceutically acceptable designation for purified Algerian bentonite, which can be used as a pharmaceutical excipient and in cosmetic products such as creams, powders and emulsions.

## Introduction

Clays are widespread in the Earth’s crust [1], and have expanded application in several engineering fields. Their applications are found in the petroleum industry, and in ceramics, paint, paper, pharmaceuticals, plastics, foundry bondants, and others [2–4]. A variety of minerals (e.g., smectite, talc, kaolinite, palygorskite and sepiolite) are among the world’s most important and useful industrial minerals, because of their high specific surface areas, optimum rheological characteristics and/or excellent sorptive capacities [3].

Since very ancient times, many clays have been extensively used in the human health industry [5]. The name bentonite was first used and proposed by Knight in 1898 [6]. Clay is formed from the alteration of pyroclastic and/or volcaniclastic rocks [7], and its main constituent is montmorillonite [8], which is a 2:1 layered silicate [9]. Indeed, bentonite is regarded as one of the most suitable materials for a large number of industries due to its advantageous characteristics, such as high cation exchange capacity and large specific surface area associated with small particle sizes [3, 10].

For pharmaceutical applications, clays, clay minerals and especially bentonite are used in pharmaceutical areas as either excipients (disintegrant agents, carriers and releasers of active ingredients, binders and diluents, emulsifying, thickening, and anticaking agents), active ingredients (antacids, gastrointestinal protectors, antidiarrhea, dermatological protectors), and cosmetic products (creams, powders, and emulsions) [11–13].

Before being used in pharmacies, bentonites must be safe. They should satisfy a number of physical, chemical and toxicological requirements [14–17]. The presence of non clay minerals (cristobalite and quartz) at levels above 2% has to be averted [12, 18], because they are reported by the international agency for research on cancer as dangerous products causing of carcinogenicity [19]. Additionally, the contents of some trace elements ( e.g., Pb and As) must be carefully controlled, and should be below the maximum required limits of pharmacopoeia for natural and beneficial bentonite (40 and 15 ppm for Pb and 5 and 3 ppm for As, respectively) [20].

In Algeria, the most important bentonite deposits are located in the northwestern part of the country, where geological reserves are estimated to be approximately 11 million tons (3.15 million tons are exploitable reserves). Currently, they are supplied by ENOF (Entreprise Nationale des Produits Miniers Non Ferreux et des Substances Utiles), with an annual production of 30,000 tons. The exploited Algerian bentonites are used in drilling muds and foundries. However, these bentonites have never been submitted to special characterization to evaluate their suitability for health care applications.

This project is intended to characterize bentonite samples from the Hammam Boughrara deposit, located in the Maghnia region, for the first time and evaluate their potential suitability in the pharmaceutical and cosmetic industries. This intended to expand use of this substance into a new field beyond its traditional scope. Bentonite materials have to completely correspond to specifications regulated by the pharmacopoeias.

To this end, the mineralogical, chemical and microbial technological features of bentonite were studied, and their microbial properties were also determined.

## Location and geological setting

**Fig. 1:**
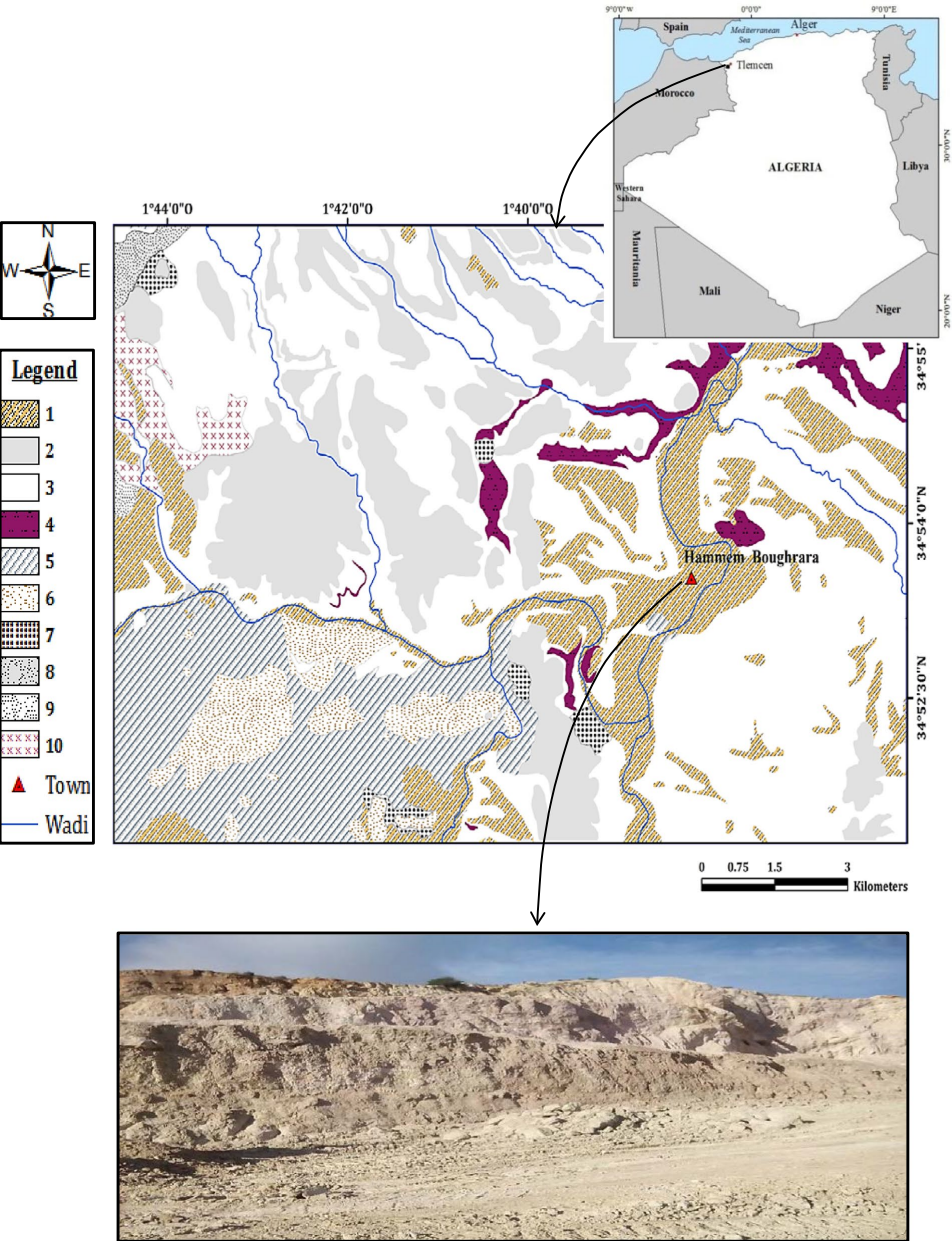
Location of the study area (Hammam Boughrara, Maghnia-Tlemcen). 1. Quaternary, 2. Pliocene, 3. Miocene, 4. Upper Miocene, 5. Upper Oxfordian, 6. Kimmeridgian, 7. Scree, 8. Dolomite, 9. Jurassic, 10. Colluvium

Our study site is named Hammam Boughrara, which is located between latitudes (34°52′ 30″N to 34°54′ 0″N) and longitudes (1°38 ′ W to 1°40′ W) in the Maghnia region, on the northwestern side of Algeria, as illustrated in Fig. 1. This region has a semicontinental Mediterranean climate and annual temperature and annual average precipitation of approximately 20 °C and 233.42 mm/year, respectively.

The region constitutes a large plain belonging to the Tafna Basin [21], which covers an approximate surface area of ~ 351 km^2^, it is elongated in an ENE -WSW direction and bounded by two Atlassic massifs; The Hercynian massif of Traras ( Fillaoussene chain) lies to the north, and the mounts of Tlemcen lies to the south [22].

The study area (Fig. 1) is geologically dominated by Miocene, Pliocene and Quaternary formations [23].

The Miocene formation is approximately 250 m in height, it is constituted of limestone, gray marl, lentils and past basaltic tuffs and basalt, and gray to greenish clays [23].

The Pliocene formation is composed of silt, sandy silt, sandy clays, sandstone and limestone, and the total thickness of Pliocene deposits varies (from 40 to 100 m) [23]. The Quaternary consists of alluvial formations that include deposits with large pebbles and blocks from ancient terraces of Wadi Tafna and some basaltic levels that appear in places. The genesis of the Hammam Boughrara bentonite deposit has been discussed by Sdran et al. in 1955 [24]. It originated from the hydrothermal alteration of rhyolite, which occurred during the tertiary marine sequence.

## Experimental

### Sample material

In the present study, a total of five fresh samples of bentonite known as RBN bentonite were collected from the Hammam Boughrara deposit. All bentonite samples were initially mixed and quartered to obtain a sample representative of the whole bentonitic deposit to extent possible.

Before use, the original bentonite was crushed into small particles, ground, sieved through a 50 μm diameter sieve, and then oven dried at 60 °C in a laboratory for a period of 24 h. Samples were conserved in desiccators until they were examined.

### Sample purification by Na + ion exchange

Purification of natural bentonite (RBN) was performed according to the procedure described by Bergaya et al. [25] with some modifications. Approximately 25 g of raw bentonite was first dispersed in 400 mL of sodium chloride solution (1 M). After about 12 h of agitation, the mixed suspension was centrifuged at 3000 r/min for 2 h. Then, the supernatant liquid was added to the same volume of 1 M NaCl. This same process (agitation + centrifugation) was repeated several times to give sodium saturated bentonite (Na^+^ bentonite).

Subsequently, the residue was washed with deionized water to remove traces of Cl^−^ ions, and again centrifuged at 4000 r/min for 3 h. The washing step was repeated until chloride ions could not be detected (as confirmed with the silver nitrate test). Finally, particles smaller than 2 μm (montmorillonite particles) were collected by sedimentation [26], and the treated sample (RBN-1) was stored in a desiccator for further experiments.

### Mineralogical and chemical analysis

The mineralogical compositions of the samples (RBN and RBN-1) were determined by X-ray diffraction techniques (XRD). XRD measurements were performed with an X'Pert Pro PANalytical diffractometer (CuKα radiation, 40 kV accelerating voltage, 20 mA intensity. The scan was recorded in the angular range of 2°–30° (2θ) with a step size 0.017° (2θ) and a scannig speed of 2° per minute.

The chemical composition (major element contents) was identified by X-ray fluorescence (XRF).The analysis was carried out on a Philips X-ray fluorescence spectrometer, model PW2400. Trace elements (Pb and Zn) were analyzed using a flame atomic absorption spectrometer (Model: Varian, AA 240FS).

### Fourier Transform Infrared Analysis (FTIR)

Fourier transform Infrared spectroscopy (FTIR) analysis was performed using a Perkin Elmer 1760 FTIR spectrometer operating in the range 4000 – 400 cm^−1^. FTIR analysis is usually used to determine the specific interactions and material compositions in composites [27].

### Scanning electron microscopy (SEM)

Morphological characteristics of bentonite particles were checked with scanning electron microscopy (SEM), using QUANTA 250 SEM equipment. SEM images were obtained with an applied acceleration voltage of 300 kV.

### CEC and SSA determinations

Cation exchange capacity (CEC) and specific surface area (SSA) were determined using the methylene blue (MB) method (spot test) [28, 29]. The CEC was computed by Eq. (1) [30]:

1 $$ {\text{CEC }} = \, \left( {{1}00 \cdot {\text{V}}_{{{\text{cc}}}} \cdot {\text{N}}_{{{\text{mb}}}} } \right) \, /{\text{ m}}_{{\text{s}}} $$

where m_s_ is the mass of the specimen (g), V_cc_ is the volume of the methylene blue titrant (mL) and N_mb_ is the normality of the methylene blue substance (meq/mL).

Specific surface area (SSA) values were calculated from the following Eq. (2) [29]:

2 $$ {\text{SSA}} = \frac{1}{319.8}\frac{1}{200} \left( {0.5{\text{N}}} \right){\text{A}}_{{\text{v}}} {\text{A}}_{{\text{MB }}} \frac{1}{10} $$

where N is the number of MB increments added to the soil suspension solution, A_v_ is Avogadro’s number, and A_MB_ is the area covered by one MB molecule.

### Particle size analysis

As with other physicochemical properties, such as CEC, SSA and SC, the surface areas of clay minerals and the small particle sizes are essential characteristics of clay that make them suitable candidates for specific pharmaceutical industries and cosmetic or skin protective applications [3, 13].

The raw and purified samples were studied by means of a laser granulometer Mastersizer (2000 Ver 4.00) to determine the range of particle sizes in our bentonites and to determine the efficiency of the purification method in separating the small particles from the bulk materials.

### Rheological properties

A suspension of 22.5 g of bentonite in 350 ml of distilled water was prepared for viscosity measurements using a FANN 35 viscometer. The reading of the viscometer at 600 rpm was taken as the apparent viscosity (A.V), while the difference between the readings taken at 600 and 300 rpm represented the plastic viscosity (P.V).

### Point of zero charge (pH _PZC_)

The point of zero charge is a very important characteristic that determines the pH at which the adsorbent surface has net electrical neutrality. pH_ZPC_ was determined using the Mular-Robert salt-pH titration method [31]. Several suspensions with a NaCl concentration of 0.001 M were prepared at various pH levels. Then, solid NaCl was added to each suspension to establish a concentration of 0.1 M. The final pH of the suspension was measured. The difference between the equilibrium pH at 0.001 M NaCl and the final pH at 0.1 M NaCl, ΔpH, was determined as a function of the final pH.

### Microbiological assessment

This part of the study was undertaken to obtain data on microbiological qualities of bentonite samples. This was determined by evaluating the presence or absence of the different pathogenic microorganisms: *Escherichia coli*, *Staphylococcus aureus*, *Pseudomonas aeruginosa*, and *Salmonella* species. The follewing experimental procedure was adapted in accordance with the requirements and the instructions of the United States Pharmacopoeia (2007) [20]. The analyses covered both untreated and treated samples.

### Total aerobic microbial count

To apply the test, a portion of ten grams (10 g) of each bentonite sample was weighed and homogenized with 100 mL of phosphate buffer ( pH 7.2). Tenfold serial dilutions of the homogenate were also prepared. The soybean-casein digest agar medium (Oxoid) was sterilized, cooled at 45 °C, and used as a culture medium. Next, one mL of each dilution was transferred to sterilized Petri dishes. Subsequently, 15 mL of culture medium was added to each plate. Then, the plates were vibrated to ensure good blending of the medium and specimens. After incubation in an inverted state for a period ranging from 48 to 72 h at a temperature of 37 °C, the number of microbial colonies (expressed as colony forming units per gram (cfu/g)) was calculated only from plates producing 30 to 300 colonies, while the other plates were discarded.

### Test for *Escherichia coli* and *Salmonella* species

The detection of total *Escherichia coli* and *Salmonella* species was performed by addition of fluid lactose medium to the specimen to establish of 100 mL, followed by incubation at a temperature of 37 °C. The medium was tested for microbial growth. Then, one milliliter portions were pipetted into vessels containing 10 ml of fluid selenite-cystine and fluid tetrathionate media, mixed, and incubated (37 °C, 12 to 24 h).

### Test for *Escherichia coli*

A mount from the remaining fluid lactose media was streaked on the surface of MacConkey agar medium plates. After incubation at 37 °C, the plates were then tested to determine the presence of characteristic colonies of *Escherichia coli*.

### Test for *Salmonella* species

Selenite–cystine and tetrathionate media were placed on the surface of bismuth sulfite agar medium and xylose–lysine–desoxycholate agar medium. After incubation at 37 °C, the Petri dishes were examined for the presence of characteristic colonies of *Salmonella* species.

### Test for *Staphylococcus aureus* and *Pseudomonas aeruginosa*

Initially, fluid soybean-casein digest medium (Oxoid) was added to each sample to obtain a volume of 100 mL, and the solution was mixed and incubated at 37 °C. Next, the contents were tested for growth, and an inoculating loop was used to streak a portion of the medium on the surface of mannitol-salt agar medium and of a cetrimide agar medium each plated on Petri dishes. After another incubation at 37 °C, the prepared plates were analyzed for the presence of *S. aureus* colonies on mannitol-salt agar medium and *P. aeruginosa* on cetrimide agar medium.

### Pharmacopeial tests

#### Sedimentation volumes (SV)

Experimental determination of sedimentation volumes was carried out according to the method described in the US Pharmacopoeia (2007). Briefly, six grams of each bentonite was first mixed with 0.3 g of magnesium oxide in 200 mL of deionized water. After blending for 5 min, 100 mL of each mixture was poured into 100 mL graduated cylindrical tubes and allowed to stand for 24 h before sedimentation volumes were measured.

#### Swelling capacity (SC)

Swelling capacity (SC) was measured according to the method reported by the US Pharmacopoeia (2007). Samples (2 g) were dispersed in 100 mL of distilled water. The dispersions were then allowed let to stand for 2 h before swelling capacity values were calculated.

#### pH

pH measurements were performed as described in the US Pharmacopoeia (2007) guidelines. Four grams (4.0 g) of each bentonite sample was dispersed in 200 mL of distilled water. Next, the obtained dispersions were mixed for 2 min before results were recorded.

## Results and discussion

### Mineralogical analysis of natural and purified bentonites

**Fig. 2:**
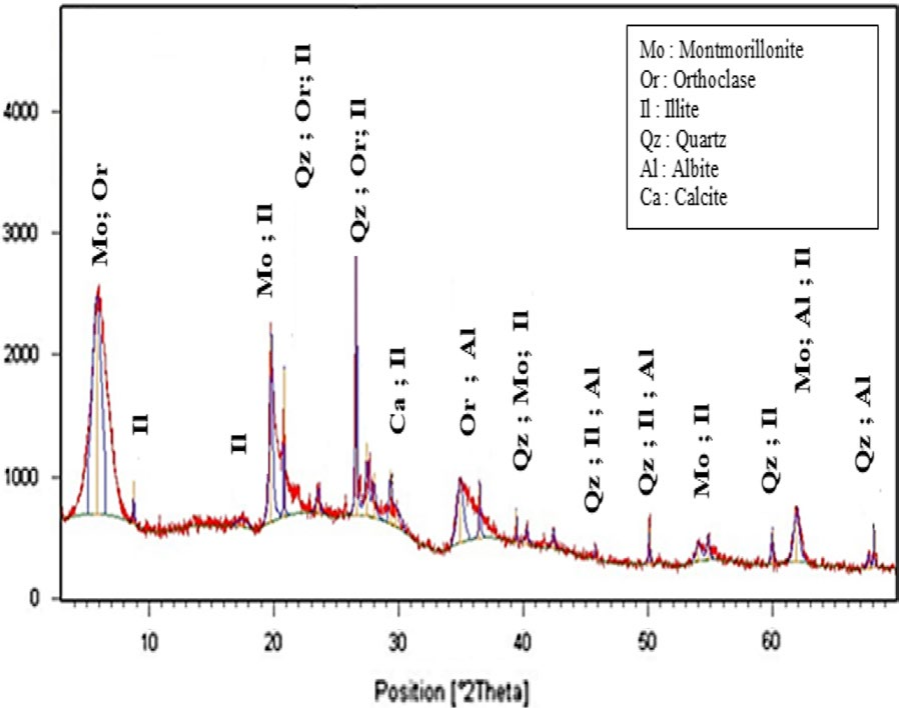
X-ray diffraction patterns of bulk bentonite RBN

**Fig. 3:**
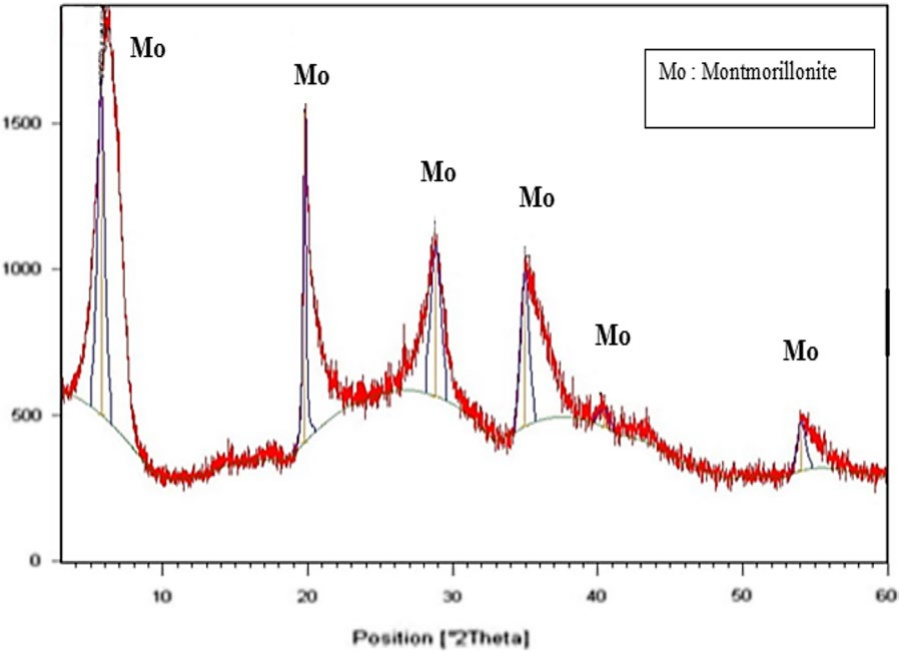
X-ray diffraction patterns of purified bentonite RBN-1

Comparative X-ray diffraction (XRD) diagrams for the RBN and RBN-1 samples are presented in Figs. 2 and 3, respectively. The mineralogical compositions of the samples clearly reveal significant differences.

XRD results shown in Table 1 indicate that the natural bentonite of the Hammam Boughrara area is mainly composed of montmorillonite (~ 59%) and illite (~ 5).The major non clay minerals are 11% quartz, 15% feldspar (orthoclase + albite) and 5% of calcite (Fig. 2). These results were not in compliance with the mineralogical compositions available in the literature for clay used in pharmaceutical and cosmetic products, due to the high amount of quartz, which was significantly higher than the allowable limits of pharmacopoeia (2%) [17].

**Table 1:** Mineral content of raw and purified samples determined by XRD

Sample	Montmorillonite (%)	Illite (%)	Feldpars (%)	Calcite (%)	Quartz (%)
RBN	59	5	20	5	11
RBN-1	100	0	0	0	0
Natural Wyoming bentonite^a^	85	0	0	5	10
Purified Wyoming bentonite^a^	100	0	0	0	0

^a^Laribi et al. [32]

XRD analysis of the bentonite sample RBN-1 after treatment showed that it was composed mostly of montmorillonite (≥ 99%). Quartz and feldspar impurities were totally absent (Fig. 3).

The absence of quartz suggested that RBN-1 has favorable characteristics for use in pharmaceutical products. The increase in montmorillonite (approximately 40%) and decrease in quartz indicated the efficiency of the sedimentation process, especially since the results obtained from purified bentonite were very similar to those of Wyoming bentonite, which is considered an ideal bentonite due to its mineral and physicochemical properties, and its suitability for various industrial applications.

### Chemical analysis

The chemical compositions of selected bentonite samples are reported in Table 2. The major element oxides found in all samples RBN and RBN-1 were SiO_2_, Al_2_O_3_ and Fe_2_O_3_, and small quantities of other compounds such as CaO, MgO, Na_2_O, TiO_2_ and SO_3_. Increased silica (SiO_2_%) and alumina (Al_2_O_3_%) contents in RBN-1 were probably associated with the low percentages of quartz and feldspar minerals which is in agreement with the XRD data [33].

**Table 2:** Chemical compositions of the raw and purified bentonite expressed as weight percent of major element oxides based on X-ray fluorescence analyses

Sample	SiO_2_ (%)	Al_2_O_3_ (%)	Fe_2_O_3_ (%)	MgO (%)	CaO (%)	Na_2_O(%)	K_2_O(%)	TiO_2_(%)	SO_3_(%)	LOI(%)
RBN	51.47	18.50	2.66	6.16	2.86	1.72	1.14	0.25	0.34	17.19
RBN-1	56.40	19 .22	2	3.11	1.56	3.12	1	0.45	0.56	12,58

*LOI* loss on ignition

The contents of CaO and MgO were low for RBN-1, which is related to the absence of carbonates. The purification process increased the content of TiO_2_ and SO_3_.

High amounts of Na_2_O were observed in the RBN-1 sample, and this difference was due to the transformation of Ca^2+^ bentonite into Na^+^ bentonite [10].

The high ignition loss (LOI) was probably due to the presence of substantial volatiles, comprised of clay minerals (mainly hydrous species), dolomite (carbonate), and organic matter [34].

All samples (RBN and RBN-1) were free of the trace elements Pb and As. The concentrations of both toxic elements were 0.04 ppm and 0.01 ppm respectively (Table 3), which are considerably lower than the allowable limits proposed by the Pharmacopoeia for "bentonite" and" purified bentonite", which are limited to maxima of 40 and 15 ppm for Pb and 5 and 3 ppm for As [20].

**Table 3:** Cation exchange capacity (CEC), specific surface area (SSA), viscosity and trace element content of raw and purified samples

Sample	CEC (meq/100 g)	SSA (m^2^/g)	Pb (ppm)	As (ppm)	Apparent viscosity(cP)	Plastic viscosity (cP)
RBN	61.76	474.64	< 0.04	< 0.01	11	7
RBN-1	88.20	677.71	< 0.04	< 0.01	36	15.5

### FTIR spectroscopy

**Fig. 4:**
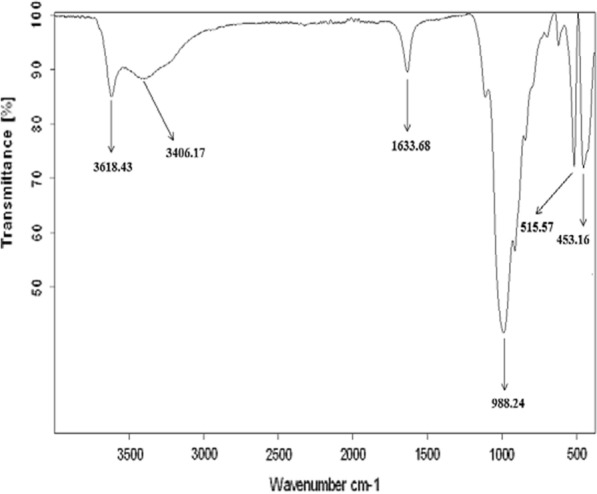
Fourier transformed infrared spectra of RBN bentonite

**Fig. 5:**
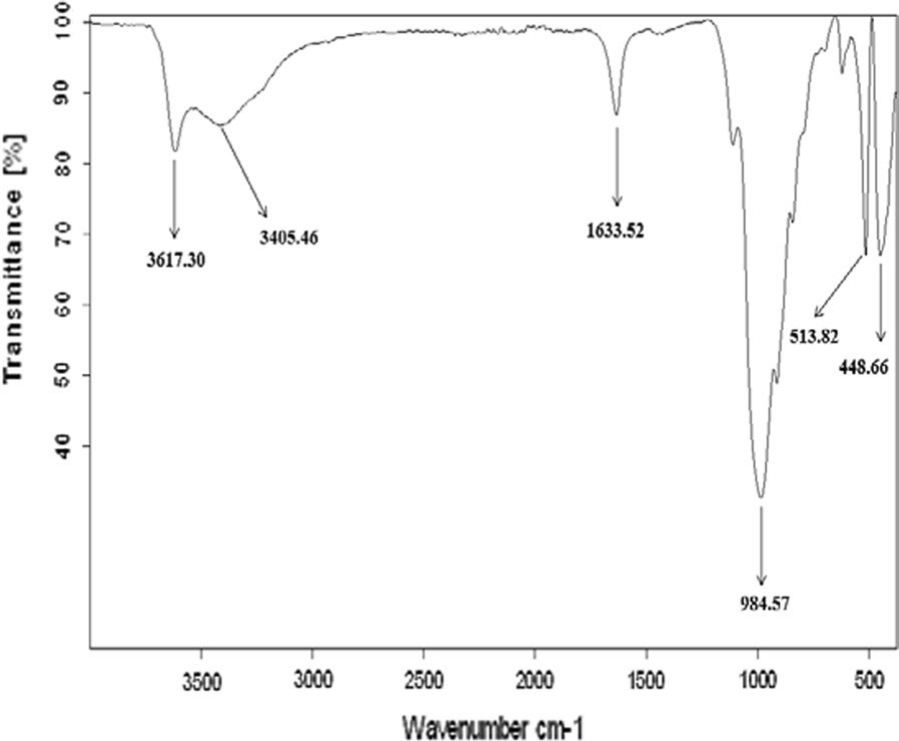
Fourier transformed infrared spectra of RBN-1 bentonite

The FTIR spectra of different bentonite samples RBN and RBN-1 are shown in Fig. 4 and in Fig. 5.The infrared spectra show the presence of stretching vibrations at 3618 cm^−1^ for structural OH groups present in the samples of clay minerals (i.e., illite, smectite, interstratified clay minerals, kaolinite and chlorite) [35]. The band near 3406 cm^−1^ is assigned to the OH stretches of water [36]. The band observed near 1633 cm^−1^ is attributed to the OH deformation mode of water [36, 37]. A broad band centered at approximately 988 cm^−1^ is ascribed to Si–O stretching vibrations [38], while Si–O–Al bending vibrations are found at 515 cm^−1^ [39, 40]. The broad band at 453 cm^−1^ is due to Si–O–Si bending vibrations [41, 42].

### SEM observations

**Fig. 6:**
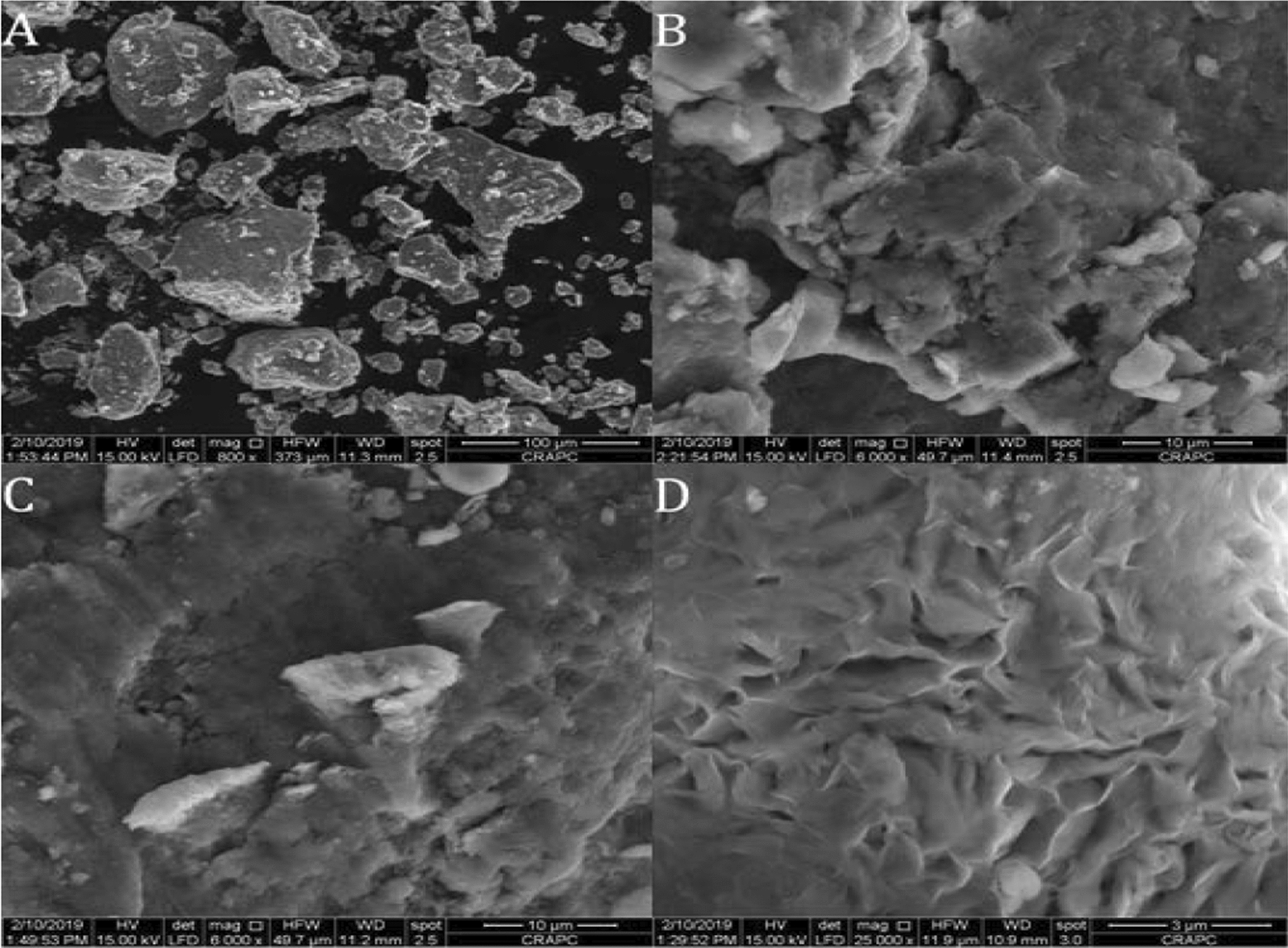
SEM images of raw bentonite RBN (A, B and C) and purified bentonite RBN-1(D)

SEM analysis was carried out to probe the effects of the purification process on the surface morphology of bentonite clay. Differences are observed in SEM micrographs for raw bentonite RBN and its treated product RBN-1. The original bentonite was composed of large pseudospherical aggregates of smectite, generally sized from 5 to 100 μm (Fig. 6A). Most of the particles were unconnected from each other. It was observed from Fig. 6B, C that the raw bentonite showed a compact and smooth morphology, which consequently diminished the surface porosity. After purification with NaCl, the surface morphology was changed significantly. However, it can be observed that the surfaces in the RBN-1 sample Fig. 6D were more porous than those in the RBN sample, which confirmed the measurements of specific surface areas (Table 3).

### Cation exchange capacity (CEC) and specific surface area (SSA)

Changes in cation exchange capacities (CEC) and specific surface areas (SSA) of raw and Na^+^ bentonite samples are illustrated in Table 3. Significant increases in SSA and CEC were observed. The cation exchange capacity ranged from 61.76 to 88.20 meq/100 g for RBN-1. This result could be related to the mineralogical changes that occurred in the clay minerals. The specific surface area increased from 474.64 to 677.71 m^2^/g after purification. Referring to Carretero and Pozo (2010) [43], these low values make the purified sample desirable for cosmetic and pharmaceutical products.

### Particle size analysis

**Fig. 7:**
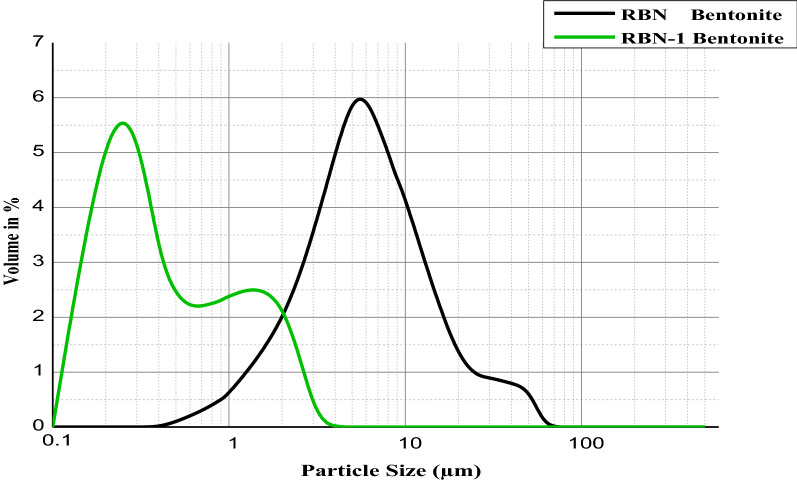
Distributions of particle sizes for RBN and RBN-1 bentonite clay

The raw bentonite sample showed a broad particle size distribution with high volume percentages centered at 5 μm and 11 μm, which were attributed to the non clay mineral particles such as quartz and feldspar (Fig. 7).

After beneficiation by NaCl, the volume percentage of the fine particle size increased dramatically compared to the raw sample, and two maxima (one centered at 0.3 μm and the other centered at 1.5 μm) were attributed to the purity of the purified bentonite clay, hence this small particle size suggests the application of purified clays in cosmetics [44].

### Rheological properties

Table 3 summarizes the apparent and plastic viscosities for the raw and purified clay samples. The A.V and P.V values ranged upward with variations from 11 to 36 cP and 7 to 15,5 cP, respectively.

These results for RBN-1 provided a clear match to the required Petrobras specification. According to the Petrobras specification, the plastic and apparent viscosity cannot be lower than 4.0 cP and 15 cP, respectively [44, 45].

### Point of zero charge (pH _PZC_)

**Fig. 8:**
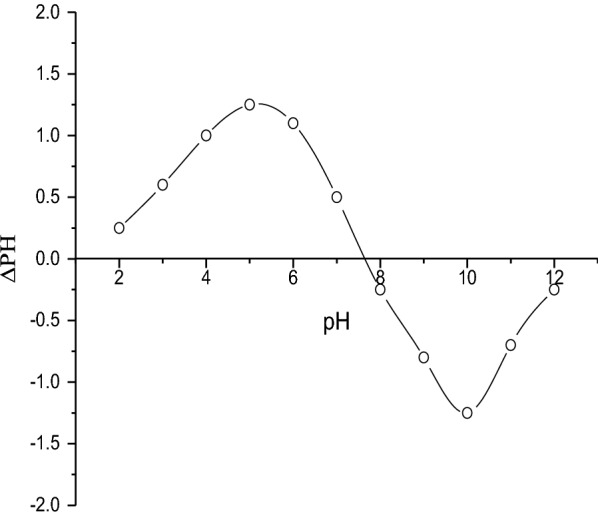
Determination of pH _PZC_ for raw bentonite RBN

**Fig. 9:**
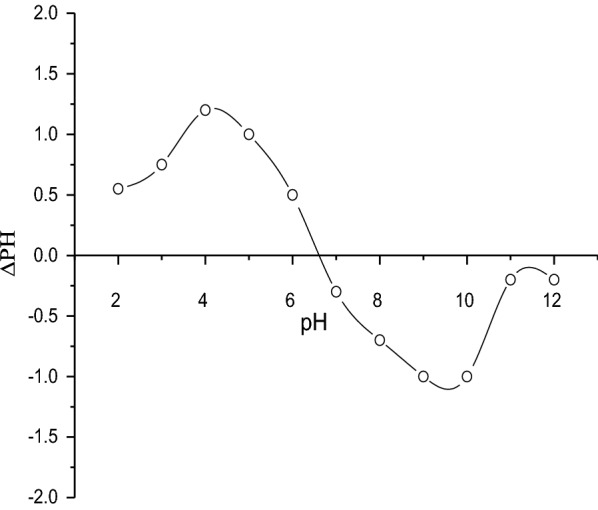
Determination of pH _PZC_ for purified bentonite RBN-1

The results of pH _PZC_ are shown in Figs. 8 and 9, and the calculated pH _PZC_ for raw and purified bentonite (RBN and RBN-1) were 7.69, and 6.61, respectively. This indicates that the treatment of bentonite increased the acidity of bentonite and made it a better adsorbent.

### Microbiological studies

The results of the microbiological tests are given in Table 4. The total microbial counts were considered in concordance with the pharmacopoeia specifications. The maximum acceptable limit for the total aerobic microbial count is 10^3^ CFU/g in nonsterile material used for pharmaceutical purposes. Pathogenic microorganisms, such as Escherichia coli, Staphylococcus aureus, Pseudomonas aeruginosa, and Salmonella were completely absent in all samples. These results confirmed the acceptable hygienic quality of these bentonite samples and guaranteed their microbiological safety.

**Table 4:** Microbial analysis (A; Absent)

Sample	Total aerobic microbial	Escherichia coli	Salmonella	Staphylococcus aureus	Pseudomonas aeruginosa
RBN	A	A	A	A	A
RBN-1	A	A	A	A	A

### Pharmacopeial tests

Table 5 presents data on the swelling power, gel formation and pH measurements of the bentonite samples. It is obvious that the pH values, sedimentation volumes and swelling capacity measurements for all the samples studied were in accordance with pharmacopoeial prerequisites.

**Table 5:** pH, swelling capacity (SC), sedimentation volumes (SV) of samples and pharmacopoeia specification

	RBN	RBN-1	Pharmacopoeia specification
pH values (2 g/100 mL)	9.12	9.30	9.0–10.5
SC (2 g/100 mL)	5 mL	25 mL	≥ 22 mL
SV (6 g/200 ml)	< 2 mL	< 2 mL	≤ 2 mL

### pH measurement

The obtained pH values ranged between 8 and 10, which made the studied clay eligible for topical use [46].

### Sedimentation volumes and swelling capacity

Sedimentation volumes were below 2 mL for each sample. Regarding the swelling volume capacity, the maximum value (35 mL) was attained after treatment and was higher than the minimum value imposed by pharmacopoeia (24 mL).

## Conclusion

In conclusion, purified Na^+^ bentonite clay was prepared through classical NaCl treatment and sedimentation, and its appropriateness for pharmaceutical use was evaluated. XRD curves showed that the raw smectitic clay was predominantly composed of montmorillonite and illite. The percentage of the montmorillonite fraction was estimated to be 100% in the purified material. Additionally, the quartz and feldspars impurities were totally removed by the purification process, which made the purified material very similar to Wyoming bentonite in their mineralogical proprieties. The results collected from chemical characterizations of all studied samples, in particular those for some harmful trace element components (Pb and As) were in accordance with the legal limits proposed by the pharmacopoeia.

The microbial content of the purified bentonite showed promising properties, which suggested its use as a pharmaceutical excipient. Purified Na^+^ bentonite could be used as a suspension agent in the formulation of topical and oral products due to its high swelling capacity, and high sedimentation volume.

Purified Algerian bentonite with a large surface area has porous particles, and a high adsorption capacity qualifies this bentonite clay for use in cosmetic products such as powders, creams and emulsions. In addition, the high CEC and SSA and good rheological properties suggest its utility as an excellent adsorbent of drugs.

## Data Availability

Data will be made available upon request.
